# Giant cell tumor of the occipital bone: A case report and review of the literature

**DOI:** 10.3892/ol.2014.2086

**Published:** 2014-04-25

**Authors:** GONCA HANEDAN USLU, EMINE CANYILMAZ, ADNAN YÖNEY, SEVDEGÜL AYDIN, ASLI ŞAHBAZ, AHMET SARI

**Affiliations:** 1 Department of Radiation Oncology, Kanuni Research and Education Hospital, Trabzon 60080, Turkey; 2 Department of Radiation Oncology, Faculty of Medicine, Karadeniz Technical University, Trabzon 61080, Turkey; 3 Department of Pathology, Faculty of Medicine, Karadeniz Technical University, Trabzon 61080, Turkey; 4 Department of Radiology, Faculty of Medicine, Karadeniz Technical University, Trabzon 61080, Turkey

**Keywords:** cranium, giant cell tumor, occipital bone, radiotherapy

## Abstract

Giant cell tumors (GCTs) are usually found in the epiphysis of the long bones, and represent ~5% of all bone tumors. Only <1% of GCTs are localized in the cranium. When localized in the cranium, GCTs are commonly observed in the sphenoid or temporal bones, and rarely in the parietal or frontal bones. Occipital bone posterior fossa involvement is an extremely rare occurrence. The current study presents a 22-year-old female patient was admitted to the Department of Radiation Oncology (Karadeniz Technical University, Faculty of Medicine, Trabzon, Turkey) with complaints of neck pain and headache. The patients cranial magnetic resonance images showed a 2.5 6-cm mass in the occipital bone, which was subtotally excised. The patient was treated with radiotherapy following the surgery. At present, the patient has shown no progression after 20 months of follow-up.

## Introductıon

Giant cell tumors (GCTs) of the bone are locally progressive and destructive borderline malignant neoplasms, which comprise ~5% of primary bone tumors and ~20% of benign tumors (1). The majority of these tumors develop in patients aged ≥20 years, with a slightly higher incidence in females. GCTs develop through endochondral ossification, and the majority of these tumors are located in the long bones of the extremities, however, a small proportion occur in the pelvis, spine or skull. Only <1% of GCTs are localized in the skull (2,3), with the most common cranial sites being the sphenoid and temporal bones.

The majority of data on GCTs in the skull consist of case reports. Despite reports of a number of cases localized in the sphenoid, temporal and parietal bones, cases of occipital bone involvement are extremely rare (1,4).

Function-preserving surgery is the standard of care for GCTs. The achievement of local control is possible in 85–90% of all cases subsequent to complete resection (5), however, in ≤50% of the cases, incomplete resection and tumor recurrence are frequently associated. Although improvements have been made to surgical techniques, certain regions, particularly the sacral or pelvic bones, the spine or the skull base, remain a challenge with regard to complete tumor removal without major functional deficits (6). As a consequence, primary radiotherapy (RT) has been recommended as an alternative treatment for GCTs in these regions; however, concerns have also been raised with regard to the local side-effects of RT at the appropriate doses (7,8).

The current study presents and discusses, with a review of the available literature, the case of a 22-year-old patient with occipital GCT who was referred to to the Department of Radiation Oncology (Karadeniz Technical University, Faculty of Medicine, Trabzon, Turkey) with complaints of neck pain and headaches. Patient provided written informed consent.

## Case report

**Figure 1:**
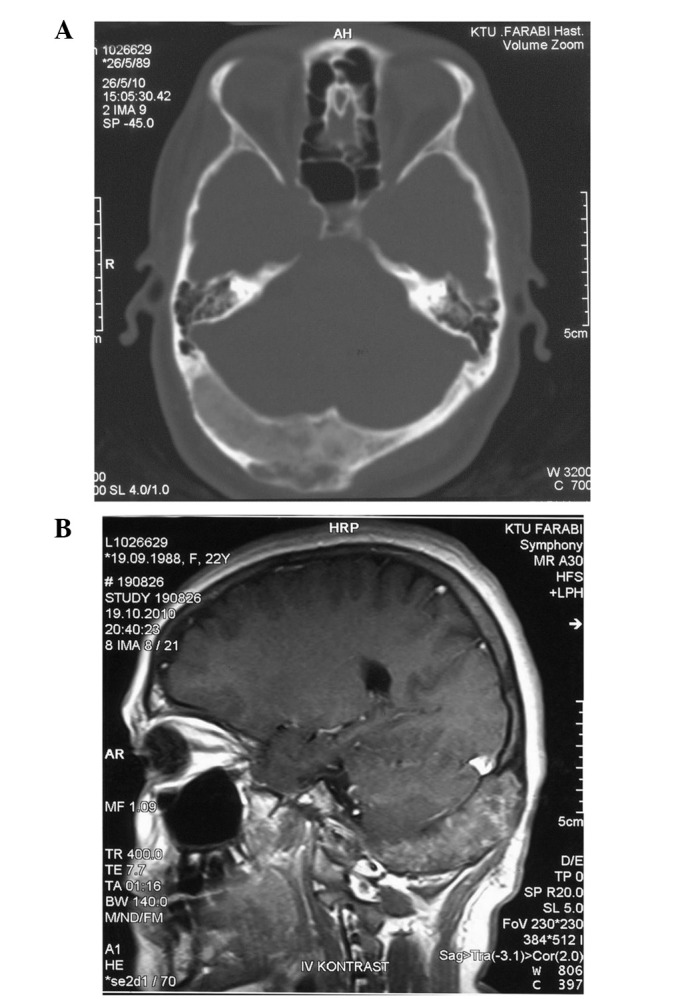
(A) Brain computed tomography revealing a ground-glass appearance within lytic areas, 2.5×6 cm. (B) Cranial magnetic resonance image showing a mass, of equal intensity with the muscle tissue, of 2.5×6 cm on the right side of the occipital bone.

A 22-year-old female patient was admitted to the Department of Radiation Oncology (Karadeniz Technical University, Faculty of Medicine, Trabzon, Turkey) with complaints of neck pain and headaches. The cranial computed tomography images images showed a ground-glass appearance with lytic areas of 2.5×6 cm in the occipital bone (Fig. 1A). The cranial magnetic resonance images showed a 2.5×6-cm mass in the occipital bone, with dural sinuses on the right side, and with the middle line slightly extending to the left. A mass that was causing expansion of the bone and that was of equal intensity with the muscle tissue in T1-weighted magnetic resonance imaging (MRI) was observed (Fig. 1B). In addition, the mass showed slightly hyperintense contrast staining in T2-weighted MRI.

**Figure 2:**
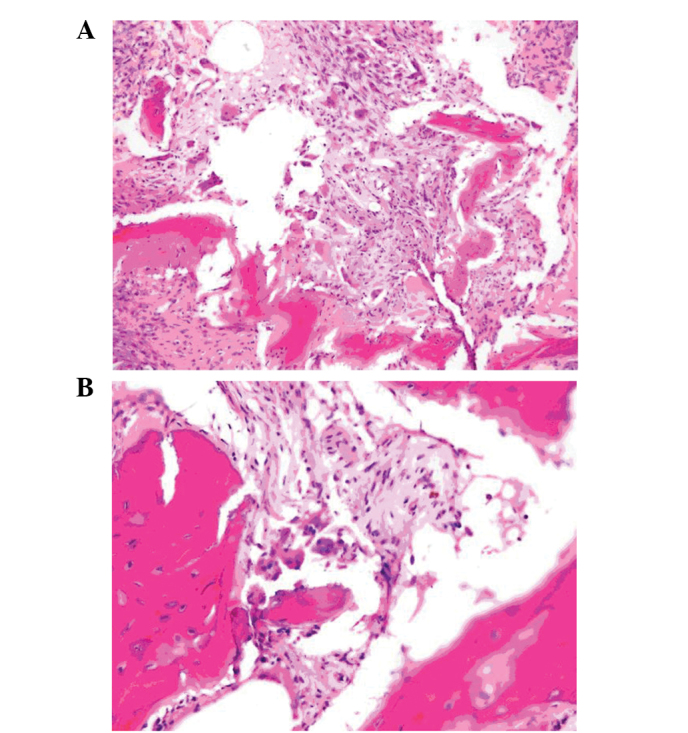
(A) Destroyed bone fragments and giant cell formations in the stroma of the neoplasm (HE stain; magnification, ×100). (B) Atypical fibrohistiocytic cells and a number of multinuclear giant cell formations were also observed; some fusiform in shape and others forming short epithelioid cells clusters (HE stain; magnification, ×200). HE, hematoxylin and eosin.

The mass was subtotally excised, and the post-operative pathological examination showed a neoplasm characterized with dispersed osteoclast-like nuclear giant cells in the fibrohistiocytic stroma between the osseous spicules. A number of foreign body-like giant cells were also detected in the neoplasm. In the immunohistochemical study, histiocytes and multinuclear giant cells were possitively stained for CD68. As a result, a GCT was diagnosed (Fig. 2).

The patient was referred to the Department of General Surgery (Karadeniz Technical University, Faculty of Medicine, Trabzon, Turkey), and post-operative RT was delivered using a 6-MV linear accelerator (Varian Clinac^®^, Varian Medical Systems, Inc., Palo Alto, CA, USA), with a 2-cm safety margin, and a dose of 50 Gy by external radiotherapy, with 200 cGy/fraction on the gross tumor volume of the subtotally resected mass. At present, the patient is being followed up and no progression has been observed for 20 months.

## Discussion

GCTs are benign, but locally aggressive, primary osseous tumors usually found in the epiphysis of the long bones (1,9), particularly involving sites such as the distal femur, proximal tibia and distal radius (10). In total, <1% of GCTs are found in the cranial bones, and typically, GCTs are observed in adults aged between 20 and 40 years (11). In an analysis across a series of GCT patients, cranial bone involvement was identified in only 24 out of 2,404 cases, the majority of which was observed in the sphenoid and temporal bones (1,7,12). In the available literature, 115 cases of GCT of the cranium have previously been reported (Table I) and a number of the case studies have reported temporal, sphenoid, frontal and parietal bone involvement (13–14). The present case is the third case of GCT of the occipital bone to be reported in the literature and the only case to undergo postoperative radiotherapy. The first case underwent excision of the mass and received no treatment following surgery. Treatment information concerning the second case was not available (Table II).

Females are affected more frequently in all age groups. The clinical presentation depends on the site of origin, however, pain and swelling in the region of the affected bone are the most common symptoms; the current patient presented with neck pain and headaches. A differential diagnosis must also consider chondroblastomas, chondrosarcomas, aneurismal bone cysts, dermoid cysts, eosinophilic granulomas and pigmented villonodular synovitis (17).

Total surgical resection is the treatment of choice for GCT, however, recurrence rates have been found to correlate with the width of the surgical excision. GCT is locally aggressive, with a recurrence rate of 40–60% (18) and the prognosis largely depends on the width of the surgical excision, as well as the radiographic and histological grading (12). Furthermore, limited evidence exists regarding the effects of chemotherapy.

The role of RT in treatment is a controversial issue. RT is an easy, safe and effective method of treatment and although no clear dose response has been identified, the literature indicates that total RT doses ranging between 35 and 45 Gy and single doses between 1.8 and 2 Gy are extremely safe and effective in controlling GCT at any location. Furthermore, total doses of >42 Gy may result in an improved outcome. RT is also effective in unresectable cases and provides a satisfactory outcome (8,19).

For those patients with GCTs that are not suitable for complete resection, primary RT must be considered as an alternative treatment method. However, this conclusion is based on data collected from small patient series over long time periods, with wide variations in radiation techniques, fractionation and total dosage. Although this treatment approach has limited available data, RT has also previously been criticized due to the low local control rates found in certain series and concerns with regard to the side-effects and induction of malignant transformation (6–8). In a series by Chakravarti *et al* (20), a 9.3-year follow-up of the patients who underwent RT was performed, and radiation-induced tumors were not observed. However, another study has argued that GCT is not radiosensitive and that it causes sarcomatous degeneration in the residual tumor tissue (21). By contrast, in two additional series, 11 out of 15 patients and 9 out of 10 patients, respectively, received adjuvant RT, and none of the patients showed sarcomatous degeneration (4,22).

Bertoni *et al* (1) showed that the use of treatment strategies involving surgical resection and RT could provide satisfactory treatment efficacy. However, in this series, total surgical excision was was the initial tratment modality and it is therefore unclear whether the RT was ultimately necessary. Coumbaras *et al* (23) and Ulu *et al* (24) each reported a case involving the cranial vault. In these cases no post-operative RT was employed and there were no signs of recurrence during the follow-up period.

In a case series by Roeder *et al* (25) concerning five patients treated with intensity-modulated RT to a median dose of 64 Gy, a local control rate of 80% was achieved. Although all primary tumors were localized in regions with directly adjacent organs at risk, including the rectum, small bowel or the optic nervous system, no severe acute or late toxicity attributable to radiation treatment has yet been observed. Furthermore, in a series of 26 lesions treated by RT at doses of 35–55 Gy, Feigenberg *et al* (26) achieved a local control rate of 77%, with three severe and four minor associated complications. Seider *et al* (27) also presented a series from the MD Anderson Cancer Center, which observed a local control rate of 70% when using doses of 36–66 Gy. The results of these series do not differ significantly, even when all patients with non-extremity tumors and those who have undergone gross total resection prior to RT have been excluded. As a consequence, the possibility of high tumor control rates can be offered by modern imaging and radiation techniques, without major side-effects.

GCTs are generally benign, locally aggressive lesions, with pain and swelling as the most common symptoms. The preferred treatment of GCT is radical surgery, and RT is restricted to inoperable cases or those not undergoing radical surgery. In the current case, RT with a total dose of 50 Gy was delivered with 200 cGy/fraction post-operatively following subtotal excision, without chemotherapy. At present, the patient is being followed up and no recurrence or symptoms have been observed for 20 months since the RT.

## Figures and Tables

**Table I:** Number of cranial region GCT cases in the literature.

Localization	Cases, n	%
Temporal bone	38	33.0
Sphenoid bone	60	52.2
Parietal bone	5	4.4
Frontal bone	9	7.8
Occipital bone	3^a^	2.6
Total	115	100.0

a One case refers to the patient of the current study.

**Table II:** Data on three patients with giant cell tumor of the occipital bone.

Case no. (ref.)	Age, years	Presenting symptom	Surgical treatment	Ancillary treatment	Outcome	Follow-up, months
1 (15)	19	Headache	Total resection	Unknown	Unknown	Unknown
2 (16)	Unknown	Neurofibromatosis	Unknown	Unknown	Unknown	Unknown
3 (Present case)	22	Neck pain and headache	Subtotal resection	Radiotherapy	Good; no radiographic progression	20
